# Facilitators and barriers of help-seeking for persons with dementia in Asia—findings from a qualitative study of informal caregivers

**DOI:** 10.3389/fpubh.2024.1396056

**Published:** 2024-07-12

**Authors:** Anitha Jeyagurunathan, Qi Yuan, Ellaisha Samari, Yunjue Zhang, Richard Goveas, Li Ling Ng, Mythily Subramaniam

**Affiliations:** ^1^Research Division, Institute of Mental Health, Singapore, Singapore; ^2^Department of Geriatric Psychiatry, Institute of Mental Health, Singapore, Singapore; ^3^Department of Psychological Medicine, Changi General Hospital, Singapore, Singapore

**Keywords:** informal caregivers, dementia, help-seeking, diagnosis-seeking, treatment-seeking, qualitative

## Abstract

**Background and aim:**

The deterioration in cognition of persons with dementia (PWD) makes their caregivers key players in their help-seeking process. This study aimed to identify the facilitators and barriers of help-seeking for persons with dementia in Asia from the perspective of their informal caregivers.

**Methods:**

A qualitative methodology was adopted in the current study. Twenty-nine informal caregivers of PWD in Singapore were interviewed between April 2019 and December 2020. All interviews were audio-recorded and transcribed verbatim for the analysis.

**Results:**

The transcripts were analyzed using inductive thematic analysis. The results revealed four major themes with 12 sub-themes, including (1) Barriers to diagnosis-seeking (i.e., lack of knowledge and awareness of dementia, emotional denial, resistance from PWD, and delays in the healthcare system); (2) Facilitators of diagnosis-seeking (i.e., synergy between awareness of dementia and an active diagnosis-seeking intention and incidental diagnosis resulting from seeking treatment for comorbid conditions); (3) Barriers to treatment-seeking (i.e., challenges from PWD and disease, challenges faced by caregivers when seeking treatment for PWD, and challenges imposed by the COVID-19 pandemic); (4) Facilitators of treatment-seeking (i.e., caregivers’ capabilities of handling PWD, cooperation/compliance from PWD, and an integrated care plan for PWD).

**Conclusion:**

The findings highlight the importance of raising public awareness, enabling health professionals to tailor psychosocial interventions better, and improving community support through dementia awareness and education.

## 1 Introduction

Dementia is a chronic and progressive syndrome that affects cognitive function and behavior among persons with dementia (PWD) (1). Currently, more than 55 million people around the world are living with dementia, and this number is expected to increase by 10 million new cases each year as the world’s population ages (2, 3), to 78 million by 2030 and 139 million by 2050 (4). According to the World Health Organization (WHO), it is the seventh leading cause of death and one of the major causes of disability and dependency among older people (3).

In comparison to other chronic conditions, dementia is a more frequent cause of care dependency among older individuals (1, 5, 6). As the disease progresses, PWD gradually lose their ability to care for themselves and independently perform activities of daily living such as bathing, household chores, and cooking. Many PWD would thus require full-time care, especially in the advanced stage, mostly from their family members (7). Studies suggest that more than three-quarters of PWD receive care in the community (8). Informal caregivers are usually the first to notice the symptoms of dementia among PWD, and therefore, they are actively involved in the help-seeking journey (9, 10). Timely diagnosis of dementia is considered of major importance to ensure adequate access to care and support for PWD. A study conducted by Eichler et al. (11) has provided evidence that routine screening improved the identification of dementia considerably and increased the diagnosis rate. Another prospective study demonstrated that PWD received a formal diagnosis through routine screening, and PWD may benefit more if they were treated with anti-dementia medications earlier (12).

The pathway to a diagnosis of dementia is believed to begin with the family’s or patient’s recognition of early symptoms, followed by active help-seeking from a primary care doctor who either diagnoses the condition or refers the patient to a specialist (13). By identifying individuals at risk of dementia in the preclinical stage and providing appropriate support and interventions, healthcare providers can help to delay or mitigate the onset of symptoms and improve outcomes for those affected by the condition. Caregivers play a dynamic role throughout the care journey and are often the primary source who encourage older adults to seek help (14). The diagnostic process can be lengthy and frustrating (15). According to previous studies, there is always a considerable time lapse between the first symptom seen or noticed and a formal diagnosis, which can be as long as 2–3 years (10, 14). The study conducted by McCleary et al. (10) explored experiences of South Asian Canadians. Early signs were attributed to aging or personality. Before seeking medical attention, family carers modified physical or social environments to accommodate the symptoms, and help-seeking was delayed up to 4 years. Another study showed that individuals may wait for 1–3 years from the onset of symptoms before receiving the diagnosis (14). A recent European study found that the average length of time lapse between PWD or their informal caregivers noticing problems and a diagnosis being made was just over 2 years (16). Receiving the diagnosis can be a shock or a relief to patients, and disclosure is an important part of management; earlier diagnosis seems to be associated with easier transitions and might delay the need for a move from home (17).

Help-seeking is a dynamic and complex process that can be influenced by many factors such as health, quality of life, treatment options, and the cost of healthcare services; and understanding help-seeking behavior for specific conditions can help to identify and reduce delays in diagnosis and treatment (18). There has been considerable research into barriers to help-seeking for dementia diagnosis over the last 30 years, with many recommending improvements in knowledge and awareness among health professionals and the public to reduce delays and facilitate diagnosis (19). Stigmatizing beliefs about dementia and inadequate knowledge were found to be the main barriers preventing people from seeking help (20). In terms of service use, various other research findings have shown that PWD and their informal caregivers use fewer services in comparison to other people in need of care (21, 22). Research suggests that the first phases of the caregiving process are critical and that the timely use of community services potentially delays institutionalization (23).

Singapore is a multiethnic, developed country in Southeast Asia, with a resident population consisting predominantly of Chinese (75.6%), Malays (15.1%), and Indians (7.6%) (24). The proportion of residents aged 65 years and above is rising at a faster pace compared to the last decade. Early diagnosis of dementia is important as this allows PWD and their informal caregivers to engage with support services and plan for the future. A study in Singapore—the Well-being of the Singapore Elderly (WiSE)—established the prevalence of dementia to be 10% among Singapore residents aged 60 years and above (25). Another study showed that the majority of the general practitioners in Singapore were positive toward the early diagnosis of dementia, endorsed the need to improve the quality of life of PWD and their informal caregivers, and preferred more training to equip themselves in the management of dementia (26). The Singapore government launched the Community Resource, Engagement and Support Team (CREST), which focuses on raising public awareness of dementia, promoting early recognition of at-risk individuals, and providing emotional support to individuals and their informal caregivers to acquire knowledge and skills to manage better (27). Despite all these efforts, studies on barriers and facilitators to help-seeking from the perspective of caregivers of PWD remain scarce. This study aimed to identify the facilitators and barriers of timely diagnosis-seeking and access to the treatment of dementia experienced by informal caregivers of PWD.

## 2 Materials and methods

### 2.1 Study design

Data for the current study were part of a qualitative project aimed at understanding the caregiving experiences of informal caregivers of PWD in Singapore.

### 2.2 Study participants

The eligibility criteria of this study were (1) Singapore citizens and permanent residents, (2) aged 21 years and above, (3) taking care of a patient who has been formally diagnosed with dementia, and (4) able to communicate in English, Mandarin, Malay, or Tamil. Those caregivers providing care to a PWD who was institutionalized in nursing homes at the point of recruitment, those who had difficulties in understanding the consent process, and the caregivers who did not visit the PWD on a weekly basis were excluded. In total, 29 informal caregivers were interviewed.

### 2.3 Data collection

Convenience sampling was employed to recruit potential participants from two sites: the outpatient clinics of the Institute of Mental Health (the sole tertiary mental health provider in Singapore) and a geriatric outpatient clinic of a general hospital (i.e., Changi General Hospital). Additionally, the study team contacted caregivers who had participated in previous research studies and had consented to future contact. Furthermore, using the snowball sampling method participants who completed the interview were invited to refer their friends who were also caregivers of PWD to join the study. The data were collected between April 2019 and December 2020, which coincided with the global pandemic caused by the novel coronavirus (COVID-19). Due to the restrictions on physical and social contact, data were collected via semi-structured interviews conducted either face-to-face or online via the Zoom platform.

### 2.4 Study procedures

Potential caregivers who expressed interest in the study were followed up by the study team via telephone to ascertain their willingness and availability to participate. Prior to the commencement of the interviews, a written informed consent was obtained from all participants. After that, participants were required to fill out a short survey on their sociodemographic information. Qualitative data were collected via semi-structured interviews. The interview guide was developed by the research team based on the literature and their experiences working with local informal caregivers of PWD, and it was reviewed by our senior researchers to ensure that it was appropriate (Table 1). Each interview was conducted by an experienced qualitative researcher using an interview guide accompanied by a note-taker (QY, YJZ, ES, and AJ). Questions covered topics including the reason for seeking help for the PWD, diagnosis, and treatment-seeking process and their feelings, and challenges while taking care of PWD. Probing questions were asked to clarify doubts and obtain valid information. Interviews continued until the research team deemed data saturation had been reached (28). The interviews were typically between 1 and 1.5 h. All interviews were audio-recorded and then transcribed verbatim with any identifying information removed. Transcripts of interviews conducted in other languages were translated into English. A total of 29 interviews were completed, among which six were conducted online via Zoom due to the COVID-19 restrictions implemented during the study period. The interviews were mainly conducted in English (QY, YJZ, ES, and AJ), while two were conducted in Chinese (QY, and YJZ). The methodology has been reported in detail in an earlier article (29). Ethical approval for this study was obtained from the National Healthcare Group Domain Specific Review Board (study reference number: 2018/01069) in Singapore.

**Table 1:** Interview guide.

Topics	Questions
Rapport building	Tell me something about yourself
	What’s your relationship with the persons with dementia (PWD)?
	How long have you stayed with the PWD?
	Or how often did you visit the PWD?
	What do you know about dementia?
	How did you get to know this information?
	When did you start taking care of the PWD?
Diagnosis-seeking	Is it before or after the formal diagnosis?
	If before—(1) When did you decide to take [the patient] to see the doctor? Or what made you think [the patient] should see the doctor?
	(2) Can you share a bit more about the process of seeking diagnosis for [the patient]?
	(3) Did you encounter any challenges or difficulties during the diagnosis-seeking process? (e.g., from family members, healthcare system, etc.). If yes, please elaborate.
	(4) How did you feel when you got to know the diagnosis of [the patient]?
	If after—(1) how did you feel when you had to take over the caregiving responsibility?
Treatment-seeking	What are the challenges and barriers you encountered during the treatment-seeking process?
	How do you think these challenges and barriers could be overcame?

### 2.5 Data analysis

The qualitative data were analyzed using an inductive thematic approach (30). This method was selected for its utility in exploring multiple perspectives, highlighting similarities and differences, providing well-structured guidelines for handling data, and identifying unanticipated insights, all of which facilitate the generation of clear and organized findings (30, 31). The data analysis comprised three iterative steps: data reduction, grouping, and abstraction (30). In particular, four transcripts were selected at random and distributed to the study team members (QY, YJZ, ES, and AJ) for repeated reading and the generation of their own codes. Subsequently, discussions were held to standardize, condense, and group these preliminary codes into a codebook with clear definitions of the codes. Multiple rounds of discussions were held for this purpose. Once the codebook had been finalized, all four team members proceeded to code three of the same transcripts in order to establish inter-rater reliability. Upon achieving a satisfactory kappa coefficient of 0.803 via NVivo Version 11, all 29 transcripts were distributed to the four researchers for independent coding.

The analysis for this study focused on identifying the barriers and facilitators associated with the help-seeking process for PWD. The lead authors systematically organized the codes related to these aspects into potential overarching themes. Subsequently, the lead authors reviewed and refined these themes. This involved evaluating whether the codes within each theme cohesively contributed to the overarching theme and assessing whether the themes accurately reflected the meanings of the data. Dependability was sustained by discussions among the team members at every stage of data collection and analysis. The team also established the study’s transferability by employing diverse research contexts (i.e., spouse caregivers and child caregivers), assuming that the findings and understandings might be generalized in other research sites. This was to ensure that we had reached thematic saturation with the data collection. All analyses were conducted using NVivo Version 11. Furthermore, in maintaining anonymity, only gender and age were included in the verbatim quotations.

## 3 Results

Twenty-nine caregivers participated in the study. The sociodemographic profile of the participants is shown in Table 2. The mean age of the participants was 56.3 years (SD = 6.5) and ranged from 46 to 72 years. The majority of participants were of Chinese ethnicity (*n* = 26, 89.7%) and daughter caregivers (*n* = 20, 69.0%). Approximately half of the caregivers (*n* = 15) had hired a domestic helper to assist in their daily caregiving activities.

**Table 2:** Sociodemographic profile of participants.

Variables		Mean (SD)	
Age (years)		56.3 (6.5)	
		***N***	**%**
Gender	Male	6	20.7
	Female	23	79.3
Ethnicity	Chinese	26	89.7
	Malay	2	6.9
	Indian	1	3.4
Relationship with person with dementia	Spouse	2	6.9
	Daughter	20	69.0
	Son	4	13.8
	Others	3	10.3
Domestic helper to support daily caregiving activities	Yes	15	52.0
	No	14	48.0

The results revealed four major themes with 12 sub-themes, including (1) Barriers to diagnosis-seeking (i.e., lack of knowledge and awareness of dementia, emotional denial, resistance from PWD, and delays in the healthcare system); (2) Facilitators of diagnosis-seeking (i.e., synergy between awareness of dementia and an active diagnosis-seeking intention, and incidental diagnosis resulting from seeking treatment for comorbid conditions); (3) Barriers to treatment-seeking (i.e., challenges from PWD and disease, challenges faced by caregivers when seeking treatment for PWD, and challenges imposed by the COVID-19 pandemic); (4) Facilitators of treatment-seeking (i.e., caregivers’ capabilities of handling PWD, cooperation/compliance from PWD, and an integrated care plan for PWD). The overview of themes and sub-themes is shown in Table 3.

**Table 3:** Overview of themes and sub-themes.

Themes	Sub-themes
Barriers to diagnosis-seeking	Lack of knowledge and awareness of dementia
	Emotional denial
	Resistance from the PWD
	Delays in the healthcare system
Facilitators of diagnosis-seeking	Synergy between awareness of dementia and an active diagnosis-seeking intention
	Incidental diagnosis resulting from seeking treatment for comorbid conditions
Barriers to treatment-seeking	Challenges from PWD and the disease
	Challenges faced by caregivers when seeking treatment for PWD
	Challenges imposed by the COVID-19 pandemic
Facilitators of treatment-seeking	Caregivers’ capabilities of handling PWD
	Cooperation/compliance from PWD
	Integrated care plan for PWD

### 3.1 Barriers to diagnosis-seeking

#### 3.1.1 Lack of knowledge and awareness of dementia

The majority of caregivers reported a lack of knowledge and awareness of dementia. While caregivers observed early changes in the behavior of the PWD, caregivers either avoided or ignored these behavior changes or could not connect these behavior changes with dementia due to a lack of awareness. Some caregivers waited until they started struggling with the PWD’s symptoms, then brought them for consultation, which, in the end, delayed the diagnosis.

*Uhh … ok … I have … personally my family do not have dementia, so I have never seen … I’m not really associated with a dementia patient. So I do not know what to expect. (P07/58/F).*

*I can recall that … I was … not sure what I should say … ridiculed by the doctor, even though at that time I did not quite feel it, that I did not understand what dementia and Alzheimer’s was all about this kind of thing. (P14/65/M).*

#### 3.1.2 Emotional denial

Emotional denial usually happens when caregivers are aware of the disease, but they refuse to admit and face the issue. This was seen in a few caregivers, where they deliberately ignored the potential disease as, to them, dementia is a ‘death sentence’ and this resulted in a delay in receiving the diagnosis.

*Err we sus … my father suspected that she’s (referring to mother) borderline demented even before she became … fully became demented. There is all these tell-tale signs. My father keep telling me “eh maybe there is some problem with her, there is some problem with her” But we sort of resisted going for proper check-up until the fall in the hospital that forced us to have the check-up anyway because my mother hates going to the hospital. (P06/68/M).*

*I was in denial, when my mom started showing signs and symptoms. Because … to me it’s a death sentence that can be very long. (P25/47/F).*

#### 3.1.3 Resistance from the PWD

Caregivers faced resistance from the PWD during the diagnosis-seeking process. Such resistance might be shown as various excuses to not visit the hospital or being uncooperative during the consultation or medical check-ups. These challenges increase the difficulties of getting a proper diagnosis for the patients and prolong the diagnosis-seeking period.

*Somehow she … she can be very resistant you know. She resisted … even the senior assistant cannot handle her. Yea even if you want to push her into that, the brain scan, you cannot, she refused to participate. (P05/62/F).*

*It took after a while because he wasn’t quite willing, it was only I think after he fell and have a fracture of … that they are able to do more, more, more, more stuff with him and all that so that then … it was only then the geriatric doctor come, come and really, come in and finally after do not know how long but he … his process is very long because of all the, the different resistance and all that kind of thing and all the different episodes. (P10/56/F).*

#### 3.1.4 Delays in the healthcare system

Another challenge faced by the caregivers was delays in the healthcare system. Many caregivers reported that the long waiting time to see a doctor was the biggest challenge, as they had to struggle with the PWD’s behavior, such as restlessness, shouting, and not being cooperative with the clinic staff during this period. Furthermore, since many tests were usually needed, caregivers might be referred multiple times to different places and must handle similar scenarios repeatedly.

*…they say you have to wait for the test, you have to wait for the scan, you have to wait for this and then yeah to me it’s long la but I do not know ah, what is the, what is the, from department to department is it long or to them it’s normal, you know what I mean? (P05/62/F).*

*…excuse me, we had to see one doctor then the doctor will refer and then after that you have to go to another place, tell the story again and then they’ll refer again. So, the problem is before you actually get the scan, you always got to see one doctor. So a lot of appointments la so that was a challenge la. (P09/53/F).*

*Yah, because partly is the appointment is every three months. So every three months for a year is about four times. four times, so that is why that every three months when they do a test the following—the next three months then we can get results. And then they—they will order another test, then another three months, then we can get the results. So at the end when they get to confirm it’s about a year time. (P16/58/F).*

In addition, some caregivers also mentioned that some clinicians seemed to lack awareness of dementia as well, which resulted in a detour in their diagnosis-seeking journey.

*when she was 57–58 that kind of thing, now she is 64. And when we went to see her former company’s doctor whom she was close with, even then the doctor also dismissed ‘no lah XXX is too young to have dementia’ that kind of thing. So we lost some time actually to the early intervention. (P14/65/M).*

### 3.2 Facilitators of diagnosis-seeking

This theme, which comprised two sub-themes, comprised caregivers’ narratives about their personal experiences and opinions that prompted them to seek a diagnosis of dementia.

#### 3.2.1 Synergy between awareness of dementia and an active diagnosis-seeking intention

The first sub-theme identified was a synergy between awareness of dementia and an active intention to seek a diagnosis. According to some caregivers, their understanding about dementia symptoms and characteristics played a crucial role in prompting them to seek help for PWD. Recognizing the behavioral changes, caregivers, armed with insightful knowledge, brought the PWD to the appropriate professionals for assistance.

*Because I noticed a few nights she was—in the middle of the night she was peeping at the door hole you know, at the opposite neighbor, and then she told us that the neighbor is watching us, keeping an eye on us, tailing us, neighbor dislike us, quite a number of times you know. So I was getting very suspicious, there was nobody opposite when you peep at that—So I told her that since she’s getting old, maybe it’s good to go for a brain check. Doctor will be able to help her, make her, help her to remember things better because now and then she forgets at that point of time so she willingly go with us and doctor confirm that she has dementia. (P24/66/F).*

For some caregivers, this awareness stemmed from significant others, such as friends or other family members, who possessed information about dementia and the pertinent help-seeking processes.

*…she (nurse friend of caregiver) came for my sister’s funeral, because we are actually family friends, so I decided that maybe I should get her advice because she’s also a nurse, right and she’s the one who told me that it could be Dementia, yes. So what I did, if I go to polyclinic, it will take time for them to get the appointment to see a doctor, right? So what I did, I called up XXX Hospital, I went direct as a private patient, yes, private so it was quite fast, much much more fast. So they gave me an appointment in less than two weeks. (P01/55/M).*

*Before that, I have actually not much knowledge about dementia. Only when my mom starts to repeat herself, ask the same question. Then I realized that maybe something is not right with her. So during one of the follow-up session at the polyclinic, I actually requested the doctor to refer her to hospital Y for a check-up. So we went for the appointment and then the doctor asked to do a MRI. And actually, from there, the results show her brain has actually shrinked, and the doctor says she is having moderate dementia. (P03/56/F).*

#### 3.2.2 Incidental diagnosis resulting from seeking treatment for comorbid conditions

The second sub-theme highlighted incidental diagnosis resulting from seeking treatment for comorbid conditions. In some cases, caregivers were following up with polyclinics (primary care clinics) or tertiary hospitals to manage the chronic physical conditions of the PWD. If any changes in behaviors were reported by the caregiver, they received immediate referrals to specialists without any delay from the treating clinicians. At other times, the treating clinicians themselves noticed the symptoms and alerted the family about the diagnosis. This proactive approach ensured that caregivers were able to get a timely diagnosis of dementia for their PWDs.

*Then when she was in the hospital, she could not quieten down. In the middle of the night, she wants to go home. Then she forgot that she has knee … she has a hip replacement, she wanted to walk … yeah … so that was when we discovered that … yah lah … you know … that she has dementia. (P07/58/F).*

*They did several test, several visits, I think may be two, three months. Previously, before that [HOSPITAL B], because we are also going [HOSPITAL B] for some other test. She was a heart centre patient there. So they did several test and then they say that she was diagnosed as having Parkinson. But also not conclusive. So we also never bother because was ok that time, so only [HOSPITAL A] confirm is dementia, then started treatment. (P13/50/F).*

*So, so then two years ago, that means like the third year, third year she got stroke again. This time it’s second time. So and then three months later she went for the routine check-up with geriatric and she failed the assessment this time, for that time so they diagnosed her with early stage dementia. (P18/46/F).*

### 3.3 Barriers to treatment-seeking

#### 3.3.1 Challenges from PWD and the disease

The first sub-theme was the challenges imposed by PWD and their disease. PWDs exhibited behavioral problems like restlessness or being loud and agitated during the treatment-seeking process, and this was usually very challenging for caregivers. In addition, some PWDs had mobility issues because of their other chronic health conditions including surgery, falls, etc. so they had difficulty going to the hospital to seek treatment. Some PWDs had difficulties expressing their needs during their consultation with specialists. They were either unable to express their issues or refused to communicate with the doctors.

*When we bring her to see a geriatrician, at this point in time she does not know how to explain. So it’s through our explanation. (P05/62/F).*

*I mean a few times the doctor had want to talk to me personally and all that kind of thing. He cannot wait outside the clinic for 5 min, he will barge in and say, why is it taking so long? Then the doctor … so got to wrap up. So it’s, it’s from going to the hospital to taking the taxi to even inside the consultation, it’s always kind of a little fights that he put up all throughout, all throughout. So, tiring. (P10/56/F).*

*now his leg cannot really walk a lot so it’s kind, somehow of leceh (troublesome) you understand … to bring him there. (P15/52/F).*

*Oh it was a real chore, because she hates the hospital right. You can see that she will cook up all kinds of stories “today I have a tummy ache you know, I do not think I can walk to the … to the taxi stand” because I do not drive, “I do not think I can take bus you know, I very headache I’ve a lot of headache,” or “I think it’s about to rain you know, better not let us not get caught in the rain, so let us not … call the hospital and say next week.” So this is the problem I face, the resistance about going to visit the hospital. (P06/68/M).*

#### 3.3.2 Challenges faced by caregivers when seeking treatment for PWD

The second sub-theme was challenges faced by caregivers when seeking treatment. The main challenges encountered among caregivers were role conflicts. Some caregivers reported difficulties in handling work/family and caregiving simultaneously. For working caregivers, they would need to take time off from their work to accompany the PWD to the hospital which might affect their work. Some caregivers mentioned the high costs during this process. A few caregivers also expressed their negative attitudes towards treatment options and made the decision regarding the medications on behalf of the PWD without consulting with the doctor.

*A lot of difficulties because I’m actually inside the operational department. I’m actually one of the main person there. So actually if I need to take time-off to attend to my family’s needs of course it will affect the operation of the company itself. (P01/55/M).*

*Ok from the medical part per se, … so many tests and they had to rule out all these so we are really not sure all the time, is this necessary, is that necessary to do*. *So it’s quite costly to have to keep on going for … and time because I’m… I have to work, I have to take care of other people as well, my children were younger then and you know in between taking care of my mom and all these things so, it was quite challenging ah. (P09/53/F).*

*So only thing she got this illness is already happen you know… the only thing that the doctor gave what sort of medicine can help her … let her eat … eat the medicine for about two-three years. My sister say that the medicine actually got side effect, so try not to take. Because take can also never get improve. So we start never let her take. Now only take (medicines for high) blood pressure and vitamin tab. (P023/56/F).*

#### 3.3.3 Challenges imposed by the COVID-19 pandemic

The third sub-theme was the challenges imposed by the COVID-19 pandemic. Some caregivers faced difficulties during the pandemic. PWDs regular follow-up appointments were changed from a few months (of regular follow-up) to yearly follow-up due to the COVID-19 pandemic. During the pandemic, PWD had to comply with several restrictions such as wearing a mask all the time in hospital or daycare settings, which was challenging for them. This led to their refusal to attend daycare centers. At the same time, a number of day care centers were also closed down, which led to a lack of social and stimulating activities for PWD.

*Emm … last time is every six to … within six to eight months like that to see the doctor. But now due to Covid-19, almost drag to almost … coming to one year. Yah … because we … because we changed the appointment due to COVID-19 lah. So it’s a bit longer … slightly longer. (P23/56/F).*

*so—because I wanted her to go to this ABC elder care. She went there for a while but when Covid started, they want clients to wear mask whole day and she does not like mask so she refuses to go there anymore. So I got to plan for her. (P24/66/F).*

### 3.4 Facilitators of treatment-seeking

#### 3.4.1 Caregivers’ capabilities of handling PWD

The first sub-theme was caregivers’ capabilities of handling PWD. Some caregivers used various strategies to bring their PWDs to clinical appointments, such as a reward system, encouraging words, and casual visits to the hospital, which reduced the agitation and anxiety in PWD.

*Oh, yeah yeah! Let say ah … that day is the appointment, so I will not tell him in advance lah. Firstly he would forget, secondly he will keep on asking again and again. So usually I will tell him on the very day itself, early in the morning lah. Then he will tell me say that what is it I need to go, I’m Ok … that kind of thing lah. He always say that I’m OK, the memory is very normal, old people also got short memory problem. (P12/63/F).*

*she is now at this stage … because I used a reward system to do … modify her behavior. So every time she goes to the doctor … 5 years down the road … every time she goes I will reward her with a lovely meal. So, she will get to eat what she likes, at the restaurant or whatever food at the hawker center. So, she will find going to the doctor very enjoyable…. (P25/47/F).*

#### 3.4.2 Cooperation/compliance from PWD

The second sub-theme was related to the PWD being cooperative/compliant. Few caregivers mentioned that their loved ones were willing to take medications and agreeable to going for various tests, assessments, etc. Some PWD had a good rapport with the doctor, so they could easily follow all the instructions given by the healthcare professionals and caregivers. Having a cooperative PWD helped the caregiver to take care of the PWD confidently and calmly during the treatment-seeking process.

*Er no, treatment, during the treatment everything is very normal. In fact when we visit her she’s very good mood one. I mean there’s a, there’s small area where you can visit and they’ll push her out, then buy her something to eat. So she’ll very good mood one. Ah there’s also struggling la I think. (P08/56/M).*

*Going to the doctor, yes, definitely compliant, he is a bit of a hypochondriac he loves going to the doctor so I got no problem with that. (P26/49/F).*

#### 3.4.3 Integrated care plan for PWD

The third sub-theme was having an integrated treatment plan for PWD and their comorbid conditions. PWD sometimes may also have other comorbid conditions; from this perspective, a treatment plan covering all the health conditions of the PWD was very helpful. It saved caregivers a lot of time and effort in managing the health of the PWD.

*OK. Previously we go to the polyclinic for her high cholesterol, her hypertension. So ever since we go to Hospital Z for this dementia, so the doctor says we can go Hospital Z and have a one-stop, no need to go to poly, which I think is also good for me. Otherwise we have to go two places you see. So now everything is all under Hospital Z, even when she do her blood test. (P03/56/F).*

## 4 Discussion

In this study, we identified facilitators and barriers to help-seeking among caregivers of PWD in Singapore. The results revealed four major themes including barriers to diagnosis-seeking (such as lack of knowledge and awareness of dementia, emotional denial, resistance from PWD, and delays in healthcare system), facilitators of diagnosis-seeking (such as awareness of dementia, incidental diagnosis resulting from seeking treatment for comorbid conditions), barriers to treatment-seeking (such as challenges from PWD, challenges faced by caregivers, and challenges imposed by COVID-19 pandemic), and facilitators of treatment-seeking (such as caregivers’ capabilities of handling PWD, cooperation from PWD and integrated care plan for PWD). Our results show that barriers to help-seeking for diagnosis and treatment do share some commonalities, such as challenges posed by PWD, long waiting times at clinics/hospitals, and lack of awareness of dementia symptoms. However, in most scenarios, they are quite distinct, and the factors to be taken into account for seeking timely diagnosis and proper treatment are quite different. Diagnosis-seeking is the period from the caregiver noticing the potential symptoms (9), initiating the diagnosis-seeking process from healthcare professionals to receiving the formal diagnosis from healthcare organizations (32). Timely diagnosis-seeking usually leads to a better follow-up and early intervention, which might significantly slow down the disease progression and thus lower the caregiving burden (33, 34). Treatment-seeking refers to the regular follow-ups and management of PWD. During this period, the deterioration is usually steady and slow (35).

Several barriers were identified which possibly led to delayed diagnosis among PWD in Singapore, including a lack of awareness among caregivers, emotional denial of caregivers, resistance from PWD, and delays within the healthcare system (19, 36–38). Given the close proximity with the PWD, caregivers are usually the first to notice the potential symptoms of dementia exhibited by PWD (9, 37). However, a lack of awareness due to a lack of knowledge of dementia and its symptoms makes it difficult for caregivers to relate these symptoms to dementia (19, 20, 38, 39). Instead, they might consider it as a normal part of aging (40), which delays the diagnosis of dementia (16). The second barrier was emotional denial. This happened when caregivers could recognize the symptoms of dementia but refused to acknowledge it and were therefore reluctant to bring the PWD for diagnoses (41–44). This is a type of avoidance coping wherein caregivers facing a dementia diagnosis may not want to accept it as it could lead to anxiety and uncertainty about the future. Such uncertainties are quite normal when individuals encounter unknown situations. However, more studies are needed to understand this better, to investigate the rationale, and to explore how to best help caregivers with such coping strategies—one possible way could be acceptance and commitment therapy training. Other than caregiver factors, resistance from PWD might also delay the diagnosis. There are several reasons for the resistance, such as a lack of understanding of dementia or the self-stigma associated with this disease (20, 45, 46). These issues highlight the need for public education on dementia, especially among older adults and those who are taking care of them (39, 47). Last but not least, the waiting time for appointments and assessments, which are delays inherent to healthcare systems, might also lead to delays in diagnosis. Even in the best-case scenario, it might still take months for PWD to receive their diagnosis (48, 49). This usually requires system-level adjustments such as organizing a separate fast lane in the current healthcare system to speed up the diagnosis process (50).

In all, three themes of barriers during treatment-seeking were identified. These included challenges from PWD, caregivers themselves, and the COVID-19 pandemic. PWD physical conditions, such as immobility, were reported to be a big challenge to caregivers, as it is very challenging to transfer them from home to the healthcare facility and back (51, 52). In Singapore, transportation services for wheelchair-bound older adults are available, however, it might not be well known among caregivers and the costs associated may be financially challenging for some of them. Considering this, telemedicine/teleconsultation might be a good alternative and the hospitals could consider introducing more of such services. Other than transportation, caregivers also reported that PWD sometimes have difficulties expressing their needs of PWDs. This becomes more obvious as the disease progresses and after PWDs gradually lose their ability to communicate (53, 54). A daily caregiving log might be helpful. However, more research is needed in this area to identify and implement better solutions. Caregivers might also encounter other issues, such as role conflicts or financial burdens (19, 49, 55). Both of these issues would require additional investments, such as providing more respite services to enable the flexibility of caregivers and more subsidies to reduce their burden. The long waiting time and the poor management of comorbidities are two other important reasons for caregivers’ reluctance to bring their PWDs for regular follow-ups (19, 43). In this case, adopting an integrated care plan comprising multiple healthcare professionals might be helpful. Last but not least, the impact of the COVID-19 pandemic was indeed quite huge on caregivers and PWD (56). However, after the high acceptance of immunization and the evolution of less detrimental mutations of the virus, it is a lesser concern right now in Singapore.

There are several practical implications of our findings. Firstly, timely diagnosis of dementia is never easy; it requires the cooperation of PWD, their caregivers, and healthcare professionals. Public awareness of dementia and its course, as well as trained healthcare professionals in the primary care setting can ensure early recognition and diagnosis of dementia (16, 33). From this perspective, more public health campaigns focusing on public education of dementia might be very helpful. Secondly, proper follow-up care of PWD, especially those with comorbidities requires an integrated care plan. Such a care plan should consider all the needs of PWDs and ensure better care quality. For caregivers, a one-stop comprehensive care plan can help them save their energy and resources, which are spent navigating across the systems, and this would lower their caregiving burden as well (57, 58). Finally, as technology advances, more and more options for remote care and remote consultation are available (59, 60). Such services are particularly suitable for informal caregivers looking after PWD for two reasons: firstly, during the treatment process, most clinicians rely on regular follow-ups to review PWD’s status and to decide whether to adjust the medications of the PWD. However, since the disease is quite stable, some of the follow-up appointments could be changed to teleconsultation and caregivers can opt for the traditional face-to-face consultation only when necessary. This would help caregivers avoid challenges such as transportation and handling PWD’s symptoms in a public area. Secondly, some digital apps can facilitate the hiring of volunteers or paid caregivers to provide short-term respite services. This could be very helpful especially when caregivers encounter emergencies. However, careful checks must be put in place by these app operators to review these volunteers or paid caregivers to ensure that they are trustworthy and capable of providing such care.

### 4.1 Strength and limitations

There are several strengths of this study. Firstly, this is the first qualitative study on barriers and facilitators of help-seeking for PWD from the perspective of informal caregivers in Asia. Sampling was purposive for characteristics that could have an impact on help-seeking for dementia and continued until theoretical saturation was reached. Data analysis was iterative and carried out independently by researchers to maximize the yield of themes and concepts. There are some limitations in this study as well. Firstly, in terms of the ethnic background of caregivers, there was an overrepresentation of Chinese ethnicity, and thus, culturally specific themes that may have emerged from the analyses were not observed. Secondly, the study participants were recruited using the snowball sampling method, and caregivers were also invited to refer their peers to join. However, this may have introduced a degree of bias, as the participants may only have been drawn from a relatively small number of caregiver circles. This may limit the generalizability of the study findings. Finally, the interviews happened only after the dyads had reached the service, findings may be affected by recall bias as the caregivers were asked to report when they first noticed symptoms and to comment on the level of difficulty of the help-seeking process retrospectively.

## 5 Conclusion

In conclusion, this study identifies important facilitators and barriers to help-seeking for PWD from the perspective of their informal caregivers in Singapore through a qualitative approach. A total of four major themes with 12 sub-themes were identified. Caregivers with sufficient knowledge of dementia seem to detect the symptoms early, hence resulting in early diagnosis and treatment. However, if caregivers do not have any knowledge and awareness, they might mistake dementia symptoms as a normal aging process. Possible solutions include an increased focus on public education, raising public awareness regarding what is and what is not a sign of normal aging, annual cognitive screening done by primary care providers, and appropriate community and healthcare support for the caregivers and the PWD both during and post diagnosis (19, 61, 62). These facilitators may overcome such barriers; however, the initiatives need to be monitored for efficacy.

## Data availability statement

The original contributions presented in the study are included in the article. Data may be available upon reasonable request and subjected to approval by the Institutional Review Board (IRB). This is a requirement mandated for this research study by our IRB and funders. Requests to access the dataset should be directed to the corresponding author.

## Ethics statement

The studies involving humans were approved by the National Healthcare Group’s Domain Specific Review Board. The studies were conducted in accordance with the local legislation and institutional requirements. The participants provided their written informed consent to participate in this study. Written informed consent was obtained from the individual(s) for the publication of any potentially identifiable images or data included in this article.

## Author contributions

AJ: Conceptualization, Data curation, Formal analysis, Investigation, Methodology, Project administration, Supervision, Writing – original draft, Writing – review & editing. QY: Conceptualization, Data curation, Formal analysis, Funding acquisition, Investigation, Methodology, Project administration, Resources, Software, Supervision, Validation, Visualization, Writing – original draft, Writing – review & editing. ES: Conceptualization, Data curation, Formal analysis, Investigation, Methodology, Project administration, Supervision, Writing – review & editing. YZ: Conceptualization, Data curation, Formal analysis, Investigation, Methodology, Project administration, Supervision, Writing – review & editing. RG: Writing – review & editing. LN: Writing – review & editing. MS: Conceptualization, Funding acquisition, Investigation, Project administration, Resources, Supervision, Writing – review & editing.
